# Special Issue “New Studies of Conjugated Compounds”

**DOI:** 10.3390/molecules25143220

**Published:** 2020-07-15

**Authors:** Yuming Zhao

**Affiliations:** Department of Chemistry, Memorial University of Newfoundland, St. John’s, NL A1B 3X7, Canada; yuming@mun.ca

For a long time, π-conjugated materials have played a central role in the fields of modern materials chemistry and nanotechnology. Ranging from fullerenes and carbon nanotubes discovered between the 1980s and 1990s [1,2,3], to molecular machines [4,5,6], organic electronic materials [7], porous functional materials (MOFs and COFs) [8,9], and the exotic carbynes and nanobelts [10,11], molecular structures with extended π-conjugated motifs are ubiquitously present. Fascinating electronic and optical properties have been found to arise from π-conjugation, making π-conjugated compounds appealing candidates for various applications. Synthetic chemistry is an underlying theme supporting the development of novel π-conjugated molecules and macromolecules, while research activities in this area have witnessed tremendous progress over the past few years.

In view of all these exciting advancements, a Special Issue, entitled *New Studies of Conjugated Compounds*, is compiled in the journal of *Molecules*, which is aimed at highlighting the recent studies in this vibrant and broadly covered field. This Special Issue contains two interesting papers. Nielsen and co-workers [12] reported on the studies of donor-acceptor substituted norbornadienes with fused aromatic (benzo, naphtho and phenanthro) groups, respectively. The norbornadiene–quadricyclane interconversion under photochemical conditions renders the system a facile molecular switch [13], which has been found to be applicable in energy storage. In this work, multi-step synthesis was conducted to produce the fused norbornadidene derivatives, in reasonable to good yields. Electronic and photochemical properties were investigated by UV-Vis and NMR analyses. It is interesting to note that the norbornadiene, after fusion with aromatic groups, loses the ability to undergo norbornadiene-to-quadricyclane photoisomerization. The findings in this work point to the fact that the photochemistry of π-conjugated compounds, unlike the ground-state reactivity, is more sophisticated and difficult to predict. Recent developments in photochemical models, such as conical intersections (CIs) [14], may offer the researchers useful tools for better understanding and visualizing the details of photochemistry.

In a mini-review article contributed by Jarosz and co-workers [15], recent advances in conjugated graft copolymers are highlighted. Copolymers are important materials in modern chemical science and engineering. Structural modifications via the “grafting strategy” present a powerful approach to generating novel polymer materials with diverse functionalities and performances. Summarizing a total of 40 articles, the authors delivered a comprehensive overview in this field. Future directions were insightfully suggested on the basis of the current progress. This review article is indeed valuable to researchers who are interested in the design and chemical modifications of organic copolymers, and the related macromolecules.

As the Guest Editor of this Special Issue, I am honored to make comments on the above-mentioned articles recently published in *Molecules*. I am very grateful to the editorial office of *Molecules*, especially Mr. Zack Li for his valuable time and support on this Special Issue. 2020 is indeed a very challenging year to many researchers, due to the global outbreak of COVID-19. Nevertheless, I strongly believe that we can and will prevail, and scientific research on π-conjugated compounds will be continually fruitful in the years to come. In closing, I would like give my sincerest thanks to all the authors and reviewers who contributed to this Special Issue, and wish everyone good health and safety in this difficult time.
